# Systemic Medication for the Relief of Sensitive Dentine

**Published:** 1896-01

**Authors:** 


ARTICLE III.

### SYSTEMIC MEDICATION FOR THE RELIEF OF SENSITIVE DENTINE.

**Read before the Washington State Dental Society by Dr. B. W. Cassil, Dayton.**

The tendency of the times seems to be toward the relief of acute pain in all operations for the relief of human ills. That this is as much true of the dental, as well as other branches of the medical profession, is apparent to all.

For this purpose, local and other anæsthetics have been introduced and given careful attention by the profession with more or less satisfactory results, where conditions are severe enough to warrant their use.

There are conditions however occurring constantly in the experience of the dental practitioner where the use of the general anæsthetic is inadmissible. In fact, as yet, no local anæsthetic or treatment of any kind has been found which would give the desired results without some injury to the organs to which they are applied, and this is sensitive dentine.

Failing in these directions to produce the desired results, attention is of necessity directed to systemic medication for relief of one of the most potent reasons for the loss of dental organs, or their neglect until the pulp lesion comcompels attention.

Much has been done by general practitioners of medicine in this direction, in conjunction with the intelligent dentists in the way of prescribing tonics, nervines, or sedatives, as the case may be, to prepare patients for the ordeal which seems to be so much dreaded by our clientele, but none so far, have recommended a specific which will act promptly, and come as near producing uniform results as salicylate of soda

Attention was first directed to the drug for use in dental practice by an article in one of the dental journals, on systemic treatment of pyorrhœa. Little attention was given the subject at the time however. A few days later a lady called to have her teeth examined, on attempting to examine a cavity on the cervical margin of a bicuspid, the dentine was found to be so excessively sensitive that she could not bear the slightest tough of an ordinary search point. An appointment was made with her for the following week, at which time after applying the dam, various local anæsthetics previously found to be more or less efficient were tried without avail in this case. Here seemed to be a case which was absolutely beyond relief, except with the forceps, the cavity being a very small one, and the set of teeth unbroken, this was not to be thought of. While considering the case, the article on Salicylate of Soda came to my mind. I began to diagnose the case from a systemic standpoint. It occurred to me as very strange that the dentine in this case should be so much more sensitive than ordinarily, unless there could be some good cause assigned for it, especially as the decay was so slight.

The writer of the article before referred to claimed that there was doubtless a systemic cause for pyorrhea which was closely allied to inflammatory rheumatism, and was produced by uric acid in the circulation.

The idea suggested to me was, that as the dentine is of similar structure to that of the bones, why should not rheumatic conditions affect the dentine as well, and be responsible for much of the soreness, therefore I asked my patient if she was subject to rheumatism and the reply was yes: "Yes, sir, more or less all the time."

Having had a good deal of experience with Salicylate of Soda in treatment of rheumatism in hospital practice in the east, and as it was nearly invariably successful, I determined to try it in this case for relief of sensitive dentine.

A prescription containing four drachms Salicylate of Soda to be taken in 15 gr. doses, four times per day was given her and she was dismissed. Four days later she returned, burs could be run at a good speed with no complaint of pain. You can rest assured that my patient was more than pleased although she complained that the medicine was rather unpleasant to take, and produced a slight nausea at times.

Such flattering success in this case determined me to continue the use of the drug whenever an operation sufficiently painful to warrant it was found.

Meeting a young girl on the street a few days after the first trial, for whom about a year previous I had been compelled to place cement fillings in the upper incisors, with very slight excavating, due to sensitive dentine, suggested her as another good subject to prove the qualifications of Salicylate of Soda, for a place in our list of remedies. She called at request and on examination the dentine was found to be as sensitive as before. The same prescription was given her with the injunction to report at once if she noticed ringing in the ears. She returned the next morning and remarked, "That stuff of yours has set a whole nest of bees going in my head," whereupon the dose was reduced to 10 grs. three times a day, and finally to 10 grs. twice per day, when ringing ceased. After the fifth day the patient was placed in the chair and the cavities excavated easily, the only complaint of pain being, at one or two points near pulp, and that but slight. Complaint being again of nausea, I desired to make a combination that would give results and still have no objectionable features if possible. Knowing Salicylate of Soda to be soluble in water, aqua peppermint was first tried, not only from the fact of its so perfectly disguising the taste of most drugs, but also for its efficiency in relief of nausea. This mixture did not quite suit me, so syrup of ginger was added to improve the flavor, as well as for its stimulating qualities.

The formula with which the best results are obtained is Salicylate of Soda 4 drachms, Aqua Peppermint 1 oz.,

Syrup of ginger 1 oz., given in teaspoonful doses, this gives us 15 gr. dose S. of S. Since using this formula there has been no complaint of unpleasant effects of any kind.

No set formuia can be given for all cases, however, one case having been met where 20 gr. four times a day for a week were used, and it may be necessary to'prescribe as much as 24 grs. per dose before getting the desired results.

This treatment is also effective for relief of facial neuralgia with almost universal success on the theory that neuralgia is due to rheumatic influences.

From my experience already with this drug I am satisfied that a remedy has been introduced that will prove invaluable in our practice, permitting a great many cases to be handled in a manner satisfactory to ourselves and our patients, which heretofore we have been obliged to patch up in a merely temporary way, to allow the patient to carry the teeth a little longer. Each of us will have to be guided by his own judgment and the conditions of the case, and satisfactory results will be assured.—*Pacific Dental Journal.*

